# Molecular Characterization and Xenotransplantation of Pancreatic Cancer Using Endoscopic Ultrasound-Guided Fine Needle Aspiration (EUS-FNA)

**DOI:** 10.3390/cancers16152721

**Published:** 2024-07-31

**Authors:** Lilia Antonova, Piriya Paramanthan, Theresa Falls, Marie-Eve Wedge, Justin Mayer, Harman S. Sekhon, John McPherson, Robert E. Denroche, Steven Gallinger, John Cameron Bell, Carolina S. Ilkow, Avijit Chatterjee

**Affiliations:** 1 Department of Otolaryngology-Head and Neck Surgery, University of Ottawa, Ottawa, ON K1N 6N5, Canada; 2 Division of Gastroenterology, The Ottawa Hospital, Ottawa, ON K1H 8L6, Canada; 3 Centre for Cancer Therapeutics, Ottawa Health Research Institute, Ottawa, ON K1H 8L6, Canada; 4 Division of Anatomic Pathology, The Ottawa Hospital, Ottawa, ON K1H 8L6, Canada; hsekhon@toh.on.ca; 5 Ontario Institute for Cancer Research, Toronto, ON M5G 0A3, Canada

**Keywords:** pancreatic cancer, xenografts, EUS-FNA, oncolytic virus

## Abstract

**Simple Summary:**

Existing treatments for pancreatic cancer have limited effectiveness due to the genetic diversity between pancreatic tumors from different patients. By propagating the original tumor into a mouse (creating a xenograft) it may be possible to study each individual tumor further and plan personalized therapy. EUS-FNA is a technique which has recently become a routine method for obtaining a biopsy from a patient without the need for surgery. In this study, we tested whether EUS-FNA can also be used to obtain tumor tissue of sufficient volume and purity to establish a corresponding xenograft and to perform a genetic analysis. We found that in the majority of cases, both mouse xenografts and genetic analyses can be performed successfully. Xenograft tumors were found to maintain the characteristics and genetic expression from the original patient tumors and to be vulnerable to infection by a virus used to destroy cancer cells.

**Abstract:**

Pancreatic cancer has one of the worst prognoses among all malignancies and few available treatment options. Patient-derived xenografts can be used to develop personalized therapy for pancreatic cancer. Endoscopic ultrasound fine-needle aspiration (EUS-FNA) may provide a powerful alternative to surgery for obtaining sufficient tissue for the establishment of patient-derived xenografts. In this study, EUS-FNA samples were obtained for 30 patients referred to the Ottawa Hospital, Ottawa, Ontario, Canada. These samples were used for xenotransplantation in NOD-SCID mice and for genetic analyses. The gene expression of pancreatic-cancer-relevant genes in xenograft tumors was examined by immunohistochemistry. Targeted sequencing of both the patient-derived tumors and xenograft tumors was performed. The xenografts’ susceptibility to oncolytic virus infection was studied by infecting xenograft-derived cells with VSV∆51-GFP. The xenograft take rate was found to be 75.9% for passage 1 and 100% for passage 2. Eighty percent of patient tumor samples were successfully sequenced to a high depth for 42 cancer genes. Xenograft histological characteristics and marker expression were maintained between passages. All tested xenograft samples were susceptible to oncoviral infection. We found that EUS-FNA is an accessible, minimally invasive technique that can be used to acquire adequate pancreatic cancer tissue for the generation of patient-derived xenografts and for genetic sequencing.

## 1. Introduction

Pancreatic cancer is projected to be the second leading cause of cancer death in the United States by 2030 [1], and it was the cause of 48,220 deaths in 2021 [2]. Since approximately 80% of pancreatic cancer patients initially present with already locally advanced or metastatic disease (non-operable), the 5-year survival for this disease is 9%, the lowest for all cancers [2]. Concerningly, little progress has been made in the treatment of pancreatic cancer in the last 30 years, and the options for second-line therapy, if primary chemotherapy fails, are limited [3]. This may be related to the high genetic diversity among pancreatic tumors, which presents a challenge to the application of a blanket treatment approach [4].

Patient-derived xenografts (PDXs) have been shown to be highly valuable for genetic and therapeutic testing in cancer studies, allowing for the application of existing and novel therapeutic options to human tumor tissue, while avoiding the exposure of patients to the related side effects [5,6,7,8,9]. The utilization of PDXs in pancreatic cancer research offers the potential for both a personalized approach to tumor characterization and treatment and the identification of tumor subtypes, aspects which would optimize the therapeutic process in a disease that is highly untreatable. While several different approaches have emerged for ex vivo pancreatic cancer research, such as the establishment of pancreatic tumor cell lines or genetically engineered mouse models, PDXs offer one of the optimal representations of the original tumor, with minimal time and resource requirements [10]. A key challenge in developing pancreatic cancer PDXs, however, is that only 15% of pancreatic cancer patients present with a disease that is surgically resectable. Previous attempts have utilized primary resected samples or tissue from tumor metastases [6]. It is likely that cancer at these stages is biologically unique and not representative of the tumor biology and subtypes found in non-resectable primary tumors [11,12]. In addition, the lack of readily obtainable tumor tissue circumvents individualized tumor characterization in the majority of pancreatic cancer patients.

Endoscopic ultrasound-guided fine needle aspiration (EUS-FNA) is a powerful alternative to surgery for obtaining biopsy samples for pancreatic cancer’s diagnosis and management [13]. This technique allows for primary tumor sampling in most pancreatic cancer patients and theoretically would present an ideal, minimally invasive method for obtaining tumor tissue for xenotransplantation. A possible limitation to this approach may be that the amount of tissue retrieved through EUS-FNA is much smaller than that obtained during surgical resection. Considering the important therapeutic and research advantages offered by the development of pancreatic cancer PDXs, we aimed to determine the feasibility of applying EUS-FNA to sample retrieval for the purpose of the genetic characterization of pancreatic tumors and the establishment of PDXs.

As existing pancreatic cancer therapeutic methods, including chemotherapy and radiation therapy, offer poor-to-moderate response rates, the exploration of novel therapeutic avenues is imperative. Encouraging results have been obtained in the treatment of other types of cancers, such as melanoma, with the use of oncolytic (cancer-lysing) viruses (OVs). These viruses have the ability to not only kill tumor cells, but also elicit potent anti-tumor immune responses [14]. A pertinent application for xenograft models of pancreatic cancer would be in the testing of such viro-therapeutic approaches for pancreatic cancer treatment.

## 2. Materials and Methods

This study was reviewed and approved by the Ottawa Health Science Network Research Ethics Board. All animal studies were approved by the institutional animal care committee of the University of Ottawa and carried out in accordance with guidelines of the National Institutes of Health and the Canadian Council on animal care.

### 2.1. Population

**Figure 1:**
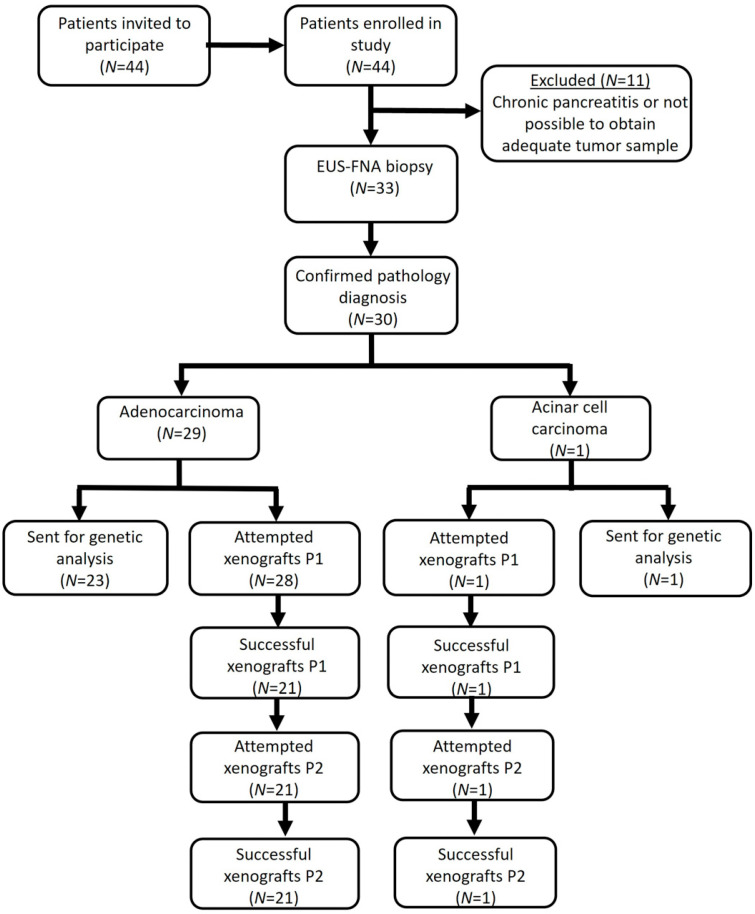
Flow diagram.

Patients referred to the Ottawa Hospital, Ottawa, Ontario, Canada, between August, 2012 and June, 2013 for a confirmatory diagnosis of pancreatic lesions suspicious for locally advanced pancreatic cancer were invited to participate in the study. The Ottawa Hospital is a tertiary referral center which provides care to an ethnically and socioeconomically diverse population. Patients diagnosed with chronic pancreatitis or for whom there were no means of obtaining an adequate tissue sample were excluded. Patient participation is described in Figure 1.

### 2.2. Sample Size Calculation

For this feasibility study, we aimed to achieve at least 10 successful xenografts and cases of genetic analysis. Approximately 90% of the tested pancreatic masses were predicted to be pancreatic cancer. Therefore, we expected that 10% of our samples would be lesions other than an adenocarcinoma. The EUS-FNA sensitivity rate is 80–90%. The xenograft take rate was projected to be ~60%, based on our previous experience with pancreatic cancer xenografts. Therefore, we estimated that 25–30 patient cases (minimum 23 cases) will be required to achieve the study’s aim.

### 2.3. EUS-FNA

Our standard diagnostic EUS-FNA protocol was followed for study-specific tumor sample extraction. Namely, the fanning technique [15] was employed, with suction, air flushing and Rapid On-Site Evaluation (ROSE) by a trained cytopathologist, using a Cook ECHO 22 g FNA needle (Cook Endoscopy, Winston-Salem, NC, USA). Two EUS-FNA samples per patient were collected, specifically for the study, into two vials of RPMI. One sample was frozen for genetic analysis within 2 h, and the other was used for tissue implantation within 24 h (Figure 1). Patients were contacted within 72 h to assess for post-procedure complications.

### 2.4. Xenotransplantation

**Figure 2:**
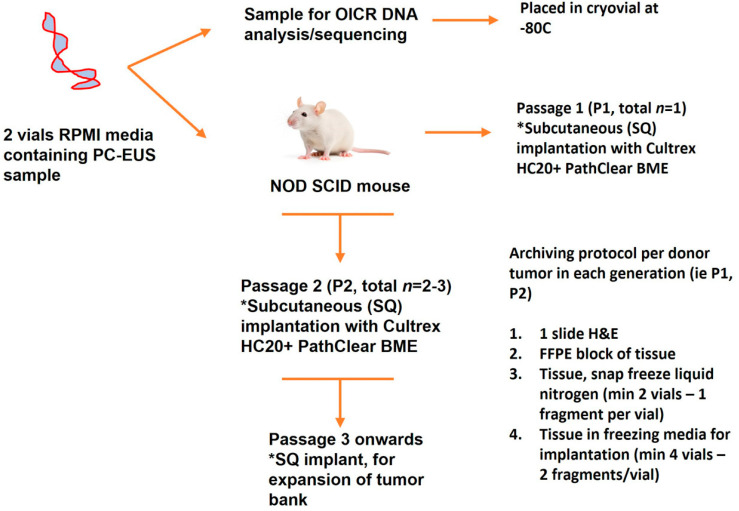
Protocol workflow. * Procedure details.

For tissue implantation, a single fresh fragment of tumor obtained by EUS-FNA was implanted subcutaneously with Cultrex® high protein concentration basement membrane extract (Trevigen, Gaithersburg, MD, USA) in 6–8-week-old nude mice (Passage 1 [P1], NOD-SCID mice, Charles River Laboratories, Wilmington, MA, USA). Their tumor growth was monitored weekly. When the tumors reached 1500 to 2000 mm^3^, they were harvested and tumor fragments were re-implanted into another cohort of mice (Passage 2 [P2]) or were frozen in liquid nitrogen in a solution of 10% dimethyl sulfoxide (DMSO) and 90% fetal bovine serum (FBS) for later use (Figure 2).

### 2.5. Sample Purity

In order to estimate the tumor cellularity of the EUS-FNA samples (the fraction of the cells in the sample which contain tumor DNA as opposed to healthy cells containing normal DNA) we considered the allele frequency of somatic *KRAS* mutations, which are present in >90% of pancreatic cancer cases. *KRAS* mutation is known to be an early event in pancreatic cancer, so we made the assumption that every cancer cell contains the event. If we also assume that somatic mutation is heterozygous in the cancer cells (and therefore affects only half of the DNA in the cells), then doubling the *KRAS* mutation allele frequency provides us with an estimate of the tumor cellularity of the sample.

### 2.6. Histologic Characterization and Immunohistochemistry of Xenograft Samples

Select xenograft passage 1 and passage 2 tumors were formalin-fixed, paraffin-embedded and sectioned before being subjected to hematoxylin and eosin staining or IHC staining. After deparaffinization and rehydration of the tumor block sections, antigen retrieval was performed in boiling sodium citrate buffer (pH 6.0). Tumor sections were stained against cytokeratin 18 (epithelial tumor cell marker, overexpressed in most adenocarcinomas) [16] (1:1000, ab55395), E-Cadherin (epithelial to mesenchymal transition marker, downregulated in advanced carcinomas) [17,18] (1:1000, ab15148), mucin 2 (intraductal papillary mucinous neoplasm cell marker, not expressed in other types of pancreatic ductal adenocarcinoma precursors) [19] (1:500, ab76774), mucin 4 (pancreatic tumor cell marker, overexpressed in pancreatic tumors) [20] (1:1000, Sigma-HPA005895) and smooth muscle actin (fibroblast marker) (1:200, ab5694).

### 2.7. Genetic Analysis

DNA from the collected samples was enriched for mutational hotspots in 42 cancer genes using a RainDance Microdroplet PCR. Barcoded Illumina libraries were constructed and then sequenced on a MiSeq instrument using a 2 × 150 paired-end protocol. Base calls were processed into demultiplexed reads with Illumina’s CASAVA software, Version 1.6 and then mapped to the hg19 human genome reference using Novoalign (v2.07.14). The Genome Analysis Toolkit (GATK v1.3.16) was employed in order to perform local realignment and base quality recalibration and to identify single-nucleotide variants. Variants that fell outside of the targeted regions, had quality values less than 500, or were found in UCSC’s repeat mask, segmental duplication or simple repeat tracks, were deemed unreliable and removed. Variant consequence (which determines whether the gene variant leads to a protein amino acid change) was predicted using ANNOVAR [21].

Several metrics and databases were used to classify variants as “likely somatic”, “potentially somatic” or “likely germline”. A variant was classified as “likely germline” if it was flagged as G5 (occurring in greater than 5% of the population) by dbSNP (https://www.ncbi.nlm.nih.gov/snp/ (accessed on 23 September 2020)), was observed in at least two normal tissue samples from other studies that used the same sequencing protocol or was observed in more than 95% of the reads (which suggests a homozygous variant that is present in both the tumor and contaminating normal fractions of the sample). The majority of the variants were classified as “likely germline”. The determining factor between “potentially somatic” and “likely somatic” variants was the status of the variant in the COSMIC database (https://cancer.sanger.ac.uk/cosmic (accessed on 23 September 2020)). Mutations that had been documented in other cancer samples were classified as “likely somatic” and the remaining novel variants were labeled “potentially somatic”.

In order to assess the potential clinical significance of the detected variants, we employed the PolyPhen-2 ([22]; http://genetics.bwh.harvard.edu/pph/ (accessed on 26 June 2024)) and SIFT ([23]; http://sift.jcvi.org/ (accessed on 26 June 2024)) databases, which provide in silico predictions of whether an amino acid change would potentially affect its corresponding protein’s structure and function. Database scores were classified as previously proposed [23,24]: (1) for PolyPhen-2—probably damaging (≥2.00), possibly damaging (1.50–1.99), potentially damaging (1.25–1.49), borderline (1.00–1.24), or benign (0.00–0.99); (2) for SIFT—intolerant (0.00–0.05), potentially intolerant (0.051–0.10), borderline (0.101–0.20), or tolerant (0.201–1.00). Blood samples were also obtained from four patients and used for targeted sequencing to establish the rate of germline and somatic mutations in the corresponding PDXs and to measure the proportion of conserved variants between the primary tumor samples and the xenografts.

### 2.8. Virus, Tumor Core Infection and Plaque Assays

The oncolytic rhabdovirus VSV∆51-GFP has previously been described [25]. When abundant tumor tissue was available, subcutaneous tumors harvested from animals were cut into 2 × 2 mm cores, distributed in 24-well dishes containing DMEM 10% FBS and infected with 1 × 10^4^ plaque-forming units (PFUs) of VSVΔ51-GFP. Pictures were taken on the EVOS FL Cell Imaging System (Thermo Fisher Scientific, Waltham, MA, USA) at a 100× magnification under fluorescent or transmitted light, as previously described [26]. After 48 h, supernatant-containing secreted viral particles from VSVΔ51-GFP-infected cores were serially diluted and titered on Vero cells as previously described [27]. The results are expressed as plaque-forming units (PFUs) per milliliter (mL).

## 3. Results

### 3.1. Patient Characteristics

Of the 44 patients who were invited to participate over a 2-year period, all consented to participate in the study. Eleven patients were excluded upon EUS as either there were no means of obtaining an adequate tissue sample, as per protocol requirements, or they were diagnosed with chronic pancreatitis. Tissue samples were collected from 33 patients, and a diagnosis of malignancy was confirmed for 30 patients (19 males, 11 females; mean age 68.9 years) (Figure 1, Table 1). Presenting symptoms included weight loss, jaundice, abdominal pain, cancer history, smoking, alcohol consumption, acute pancreatitis and diabetes. Ninety-seven percent of the pancreatic masses were diagnosed as ductal pancreatic adenocarcinoma, as confirmed by a pathologist (*n* = 29). One patient’s pancreatic mass was diagnosed as an acinar cell carcinoma (*n* = 1). Of the pancreatic adenocarcinomas, 58.6% (*n* = 17) of the samples were well differentiated, 34.3% (*n* = 10) were moderately differentiated and 6.9% (*n* = 2) were poorly differentiated.

### 3.2. EUS-FNA

EUS-FNA was performed as part of the standard of care for an initial pancreatic cancer diagnosis. The number of EUS-FNA passes required for diagnosis ranged from 3 to 5. Two EUS-FNA passes were made for implantation in mice and subsequent PDX generation. The average cell count yielded from the two EUS-FNA passes was 396.2 ± 137.7. No complications were reported after EUS-FNA for any of the 30 patients.

**Figure 3:**
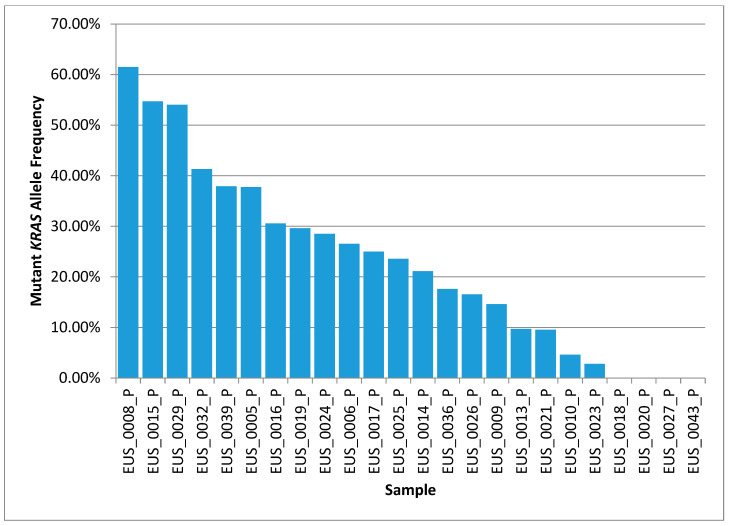
Mutant *KRAS* allele frequency results from the 24 tumor samples that underwent deep, targeted sequencing. The tumor cellularity of the samples can be roughly estimated by doubling the allele frequency.

Figure 3 displays the allele frequency of somatic *KRAS* mutations in the 24 patient samples that were successfully sequenced. Three of the samples show frequencies of greater than 50%, which suggests that copy number amplification of the *KRAS* locus has occurred in these samples and that simply doubling the *KRAS* frequency will overestimate the tumor’s cellularity—a limitation of this approach. With that caveat in mind, we estimate that the tumor cellularity of the 24 samples ranges from 0% to 80%. We did not detect *KRAS* mutations in four of the samples, either because they were *KRAS* wild-type or because there was no detectable tumor material in the sample. Based on the other somatic variants observed, we estimated that 2 of the 4 samples without detectable *KRAS* did contain tumor material at a useful cellularity.

### 3.3. Generation of a Pancreatic Tumor Graft Biobank

**Figure 4:**
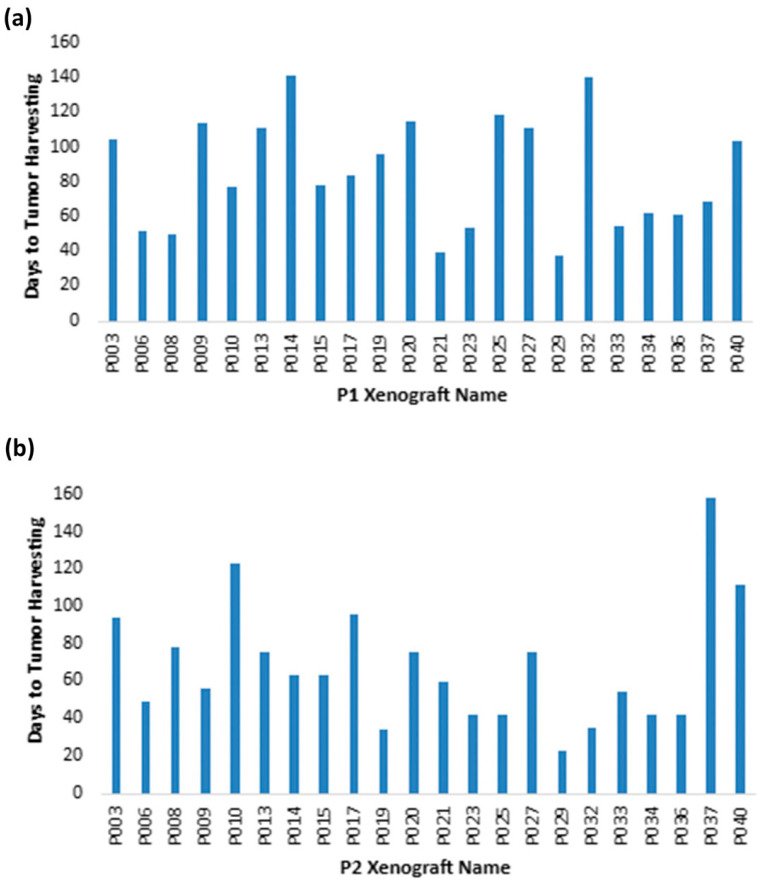
Time to tumor harvesting for xenograft passages (**a**) P1 and (**b**) P2.

In total, this study included 29 attempted patient grafts with confirmed primary tumor diagnosis, implanted subcutaneously in the flanks of NOD/SCID mice. One sample was excluded due to insufficient starting tissue material. For all samples with true subcutaneous tumor growth, the xenograft take rate was 75.9% (22/29 attempted) for passage 1 (two samples were found to be tissue other than pancreatic, and five samples did not show growth) and 100% (22/22 attempted) for passage 2. The average tumor diameter was 1.2 cm and the average number of tumor growth days prior to the defined endpoint was 85.4 ± 13.0 days for P1 and 68.0 ± 13.43 days for P2 (Figure 4). Percent tumor area was estimated by a blinded pathologist for 81.8% of the P1 xenografts and 31.8% of the P2 xenografts (Supplementary Table S1). The mean tumor area percentage was 73.1 ± 8.4 and 65.0 ± 14.9, respectively.

### 3.4. Genetic Sequencing Feasibility

The DNA from 24 samples out of the 30 attempted was successfully enriched for 42 cancer genes and sequenced to a high depth (with an average of >2000× coverage). Single-nucleotide variants were identified from this targeted sequencing approach using GATK (Version 4.2 (accessed on 25 October 2020)) and the classification system described in the Methods section. An average of 32.3 variants were identified in each sample, with 2 “likely somatic” and 0.92 “potentially somatic” variants labeled per sample, on average (the remaining variants were classified as “likely germline”).

Based on the *KRAS* mutation-derived estimates of tumor cellularity, roughly 14 of the 24 samples would be suitable for whole-exome sequencing, and only 3 of the samples would be ideal for whole-genome sequencing.

### 3.5. Patient-Derived Xenografts Express Pancreatic Cancer Markers and Retain Their Histological Characteristics after Passaging

**Figure 5:**
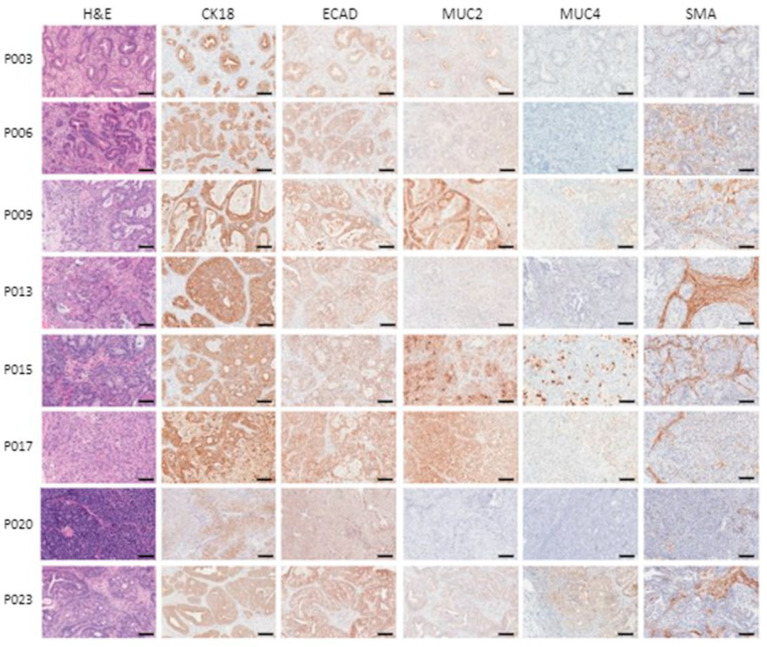
Histology and immunohistochemical analysis of patient-derived xenografts. Hematoxylin and eosin staining of several xenograft samples and IHC images of tumor blocks stained with cytokeratin 18, E-Cadherin, mucin 2 and 4 and smooth muscle actin. Row labels denote sample designation. Scale bars are equal to 100 µm.

**Figure 6:**
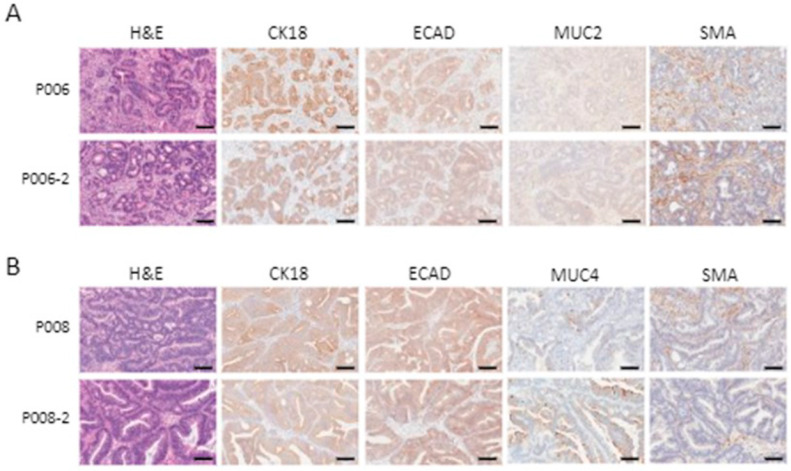
Patient-derived xenografts maintain their histological characteristics and cellular marker expression after passaging in mice. Two representative xenografts shown after the initial graft (P006 and P008) and after an additional passage in mice (P006-2 and P008-2). Conserved histology and phenotype are shown using H&E staining and the IHC processing of samples with cytokeratin 18 (CK18), E-Cadherin (ECAD), mucin 4 (MUC4) and smooth muscle actin (SMA). Scale bars are equal to100 µm. (**A**) Sample P006. (**B**) Sample P008.

Xenograft tissue staining showed the expression of markers found in some types of pancreatic cancers (ECAD, MUC2 and MUC4), as well as of the pan-cancer cytokeratin CK18, to various degrees (Figure 5). Histological characteristics and marker expression were maintained after passaging the tissue in mice (Figure 6).

The genetic sequencing analysis of patient samples identified a number of genetic variations in cancer-relevant genes. These were classified as likely germline, likely somatic or somatic, using the techniques explained in the Methods section. The genetic variations considered to be spontaneously arising mutations (somatic) are listed in Table 2. Such variations were observed in several tumor suppressors and protooncogenes and were often found in more than one tumor sample, with the highest rate of somatic mutations occurring in genes coding for the tumor suppressors MLH1, TP53 and APC and the oncogenes Notch1 and BRAF. Somatic mutations were run through the PolyPhen-2 and SIFT databases to assess their potential functional significance. Fourteen percent of variants were recognized in PolyPhen-2 and 38% in SIFT. Of the recognized variants, certain mutations in TP53, APC, MET and PIK3CA were assessed as being potentially deleterious (Table 2).

To confirm whether the somatic genetic variations observed in xenograft sequencing originated in the corresponding primary tumor, targeted sequencing was carried out on four primary tumors and xenografts, using non-malignant tissue from the patient as control. The four sets of samples included three tumors diagnosed as adenocarcinoma and the tumor diagnosed as acinar cell carcinoma. Each of the three adenocarcinomas demonstrated somatic KRAS mutations which were maintained between primary tumor and xenografts (Table 3). One of the samples contained a TP53 mutation conserved between the primary tumor and xenograft. KRAS mutations were not observed in the acinar cell carcinoma, consistent with the acinar cell pancreatic cancer genotype. All observed somatic mutations had been projected to be likely somatic using the classification described in the Methods section.

### 3.6. Patient-Derived Xenograft Models Can Be Used to Evaluate Oncolytic Virotherapy

**Figure 7:**
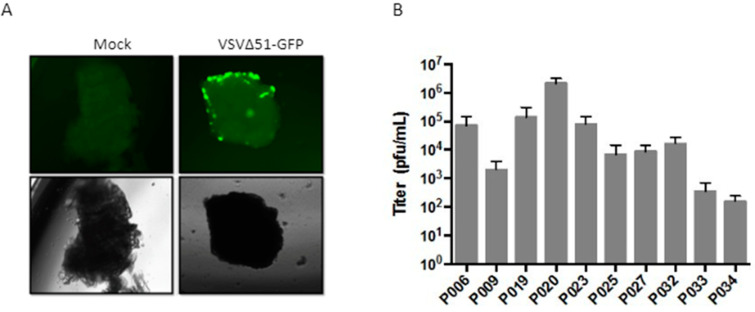
Ex vivo infection of patient-derived xenograft core samples. Tumor cores were infected using 1 × 10^4^ plaque-forming units (PFUs) of VSVΔ51-GFP. After 48 h, tumor cores were imaged under fluorescent light or transmitted light (100X). (**A**) P006 xenograft infection imaging. (**B**) Secreted viral titers at 48 h post-infection.

The susceptibility of the established PDXs to oncolytic viruses, such as a VSV∆51-GFP infection, was evaluated by performing ex vivo tumor tissue infections on 10 xenograft samples. Although the results show different infectivity levels amongst patients, ranging from 10^2^ to 10^6^ pfu/mL, all tested samples were susceptible to OV infection (Figure 7). Interestingly, the acinar cell tumor xenograft showed the highest level of oncolytic virus infectivity.

## 4. Discussion

In the study presented here, we demonstrate that EUS-FNA can be successfully used in place of traditional surgical biopsy to obtain a high-quality tumor sample in a sufficient quantity for establishing mouse xenografts, as well as for genetic tumor characterization. Using samples obtained by EUS-FNA, we were able to establish a working bank of serially transplantable pancreatic tumor grafts that retain the characteristics of the original tumor. This tumor bank provides valuable pre-clinical models for investigating personalized anti-pancreatic cancer therapies, such as the administration of oncolytic virus treatments.

To our knowledge, only two other studies to date have investigated the possibility of applying EUS-FNA to the mouse grafting of pancreatic tumors for functional therapeutic studies. In a study by Berry and colleagues, two patient-derived xenografts were established for the purpose of evaluating the response of KRAS wild-type tumors to the EGFR inhibitor panitumumab [28]. The authors found that xenograft tumor growth was successfully inhibited in response to chemotherapy. However, an overall graft success rate was not reported. This and the small number of attempted grafts make it difficult to evaluate whether those findings are comparable to ours in terms of graft establishment. A study by Allaway and colleagues of 24 primary tumor EUS-FNA-derived xenografts reported a success rate of 37.5% and a successful passage of the majority of xenografts up to F5 [29]. Here, we report a much higher success rate for the establishment of first-generation patient-derived xenografts, 75.9%, and we were able to successfully establish 100% of our second-generation xenografts.

Conceivably, particular aspects of the applied EUS-FNA technique are likely to highly affect sample integrity and any subsequent xenograft development. In this study, we chose to adhere to our diagnostic EUS-FNA protocol during sample acquisition in order to test the technique’s effectiveness under routine clinical conditions. Our diagnostic protocol is built on recommended aspects of fine needle aspiration. For instance, the addition of the suction technique has been shown to improve diagnostic yield, accuracy and sensitivity [30]. Similarly, the fanning technique, involving sampling multiple regions within a lesion in a single pass, has been shown to be superior to the standard technique due to the reduced number of passes required [15]. Air flushing was employed whenever possible, as it has been proven an easier and safer technique [30]. Finally, the use of ROSE during EUS-FNA is thought to improve EUS-FNA’s sensitivity and adequacy rate [31]. While continued research may change the relevance of these parameters, our work demonstrates that this particular set of techniques, when employed by an experienced endoscopist, can produce samples with sufficient purity and volume for the successful establishment of patient-derived xenografts. Contrast-enhanced harmonic EUS-FNA is a newer method that has been shown in a recent meta-analysis to improve diagnostic accuracy and sample adequacy compared to standard EUS-FNA [32]. However, its applicability in the context of obtaining tissue samples for the purpose of xenograft generation and genetic analysis has not been examined and may present an intriguing future avenue of investigation.

Berry and colleagues also evaluated the feasibility of obtaining material for genetic analysis in a larger cohort of 40 patients. They were highly successful in obtaining sufficient genetic material for targeted RNA sequencing from EUS-FNA samples. We similarly found that while less than 50% of samples have sufficient tumor material to perform exome-wide DNA sequencing, in 80% of cases, EUS-FNA could be successfully used to derive genetic material of sufficient quantity and purity for targeted DNA sequencing. The latter type of sequencing is sufficient for identifying pancreatic tumor subtypes previously defined based on their response to various therapeutic agents [28], thus personalizing therapy in accordance with known therapeutic profiles.

While a targeted sequencing approach may be sufficient for many tumors and preferable if high tumor cellularity cannot be achieved during EUS-FNA (a reduced depth of coverage during genome-wide sequencing may result in a loss of sensitivity for mutation detection tools), the identification of novel tumor subtypes would require a wider-spanning sequencing approach. Pancreatic cancer’s molecular and histological subtypes are still not clearly defined and debated in the literature, making their identification a key step towards precision medicine [33]. For the purpose of large-scale exome sequencing, the tissue yield can be enhanced with additional EUS-FNA needle passes. In the study by Berry et al., one additional EUS-FNA pass produced a 10-fold improvement in genomic DNA yield [28]. Increasing the number of EUS-FNA passes, however, may also result in an increase in the risk of procedural complications [34]. Alternatively, the tissue yield may be improved by the application of emerging technologies for EUS-based tissue acquisition, such as fine-needle biopsy. A pilot study on EUS-FNB reported a patient-derived engraftment rate of 60% [35].

Most pancreatic ductal adenocarcinomas continue to be diagnosed by EUS-FNA; with no clear guidelines for EUS-FNB’s use, EUS-FNA is the standard of care for sampling pancreatic solid masses, subepithelial lesions and lymph nodes recommended by the American Society of Gastrointestinal Endoscopy (ASGE) and the European Society of Gastrointestinal Endoscopy (ESGE) [36,37,38]. Therefore, despite the particular challenges related to genome-wide genetic analyses, the application of EUS-FNA to tumor tissue acquisition in the course of xenograft generation may represent the most relevant clinical scenario. Some pancreatic cancer-specific meta-analyses [39,40] and RCTs [41,42] have demonstrated similar diagnostic adequacy and core specimen procurements for EUS-FNA and EUS-FNB, as well as superior acquisition of next-generation sequencing material in the case of EUS-FNA [43].

Patient-derived xenograft models have previously been shown to accurately represent human disease at the histological and genomic levels [44,45,46], which we also show in this study. These models can, thus, serve as a promising tool to evaluate the effectiveness of novel and existing pancreatic cancer therapeutics. The targeted genetic sequencing of our established xenografts identified a number of somatic mutations in genes important for the development of pancreatic cancer, which may be potential therapeutic targets. In addition to the expected KRAS mutations, which occur early in pancreatic cancer’s development and are found in more than 90% of pancreatic tumors, mutations were most frequently observed in the tumor suppressor genes MLH1, TP53 and APC, as well as the oncogenes Notch1 and BRAF. BRAF lies downstream of KRAS signaling in activating the transcription factors c-Jun and ELK1 and promoting cell proliferation. BRAF mutations are relatively rare in pancreatic tumors, previously observed at a frequency of 2% [47] and occurring at a rate of 6% in our patient population. Importantly, combination therapy involving RAF and MEK pathway inhibition has become the standard of care for melanoma cancers demonstrating BRAF mutations [48]. Therefore, whereas generalized KRAS targeting has proven to be largely unsuccessful clinically [49], one example of advancing precision medicine through the use of EUS-FNA-derived xenografts could be the isolation of BRAF-mutant tumors and the testing of the RAF/MEK therapeutic strategy on such tumors.

p53 is one of the most commonly mutated genes in human cancers, with mutations having been previously observed at a rate of 60% in pancreatic tumors [47]. Sixteen percent of the observed mutations in our tumor bank that were predicted to be somatic were in the p53 gene. Three different TP53 mutations were recognized by PolyPhen-2 and/or SIFT and assessed as being deleterious. While various strategies targeting p53 are currently being investigated, they are still in their early clinical trial stages [50]. In vitro studies have shown a negative effect of the loss of p53 function on pancreatic cancer chemotherapy responsiveness [51]. Conversely, a recent study demonstrated that p53 mutations in pancreatic cancers conferred sensitivity to adjuvant gemcitabine, the most commonly applied therapeutic agent [52]. The relationship between p53 status and existing therapeutics could be further examined with the use of pancreatic cancer xenografts.

Interestingly, one of the primary tumors included in this study was diagnosed as a rare pancreatic acinar cell carcinoma (PACC). This type of exocrine tumor accounts for only 1% of pancreatic cancers. It is generally associated with a better long-term prognosis than that of pancreatic adenocarcinomas; however, no clear treatment guidelines exist due to the scarcity of occasions for observing the therapeutic effectiveness of different therapies. Opportunities to characterize PACCs and develop therapeutic strategies, such as can be found with the use of patient-derived xenografts, are highly valuable. In our study, this type of tumor stained differently than adenocarcinomas for CK18 and exhibited mutations for Notch 1 and APC. Inactivating mutations in genes of the adenomatous polyposis coli (*APC*)-β-catenin pathway have been previously reported in up to 25% of PACCs [53]. Consistent with previous reports, the PACC in our study lacked mutations in the KRAS gene [54]. Significantly, in addition to being highly amenable to xenograft generation, this tumor demonstrated the highest rate of viral infection among all tested samples, suggesting a potential therapeutic avenue for this type of pancreatic malignancy.

Recent advances in the use of oncolytic viruses have provided new therapeutic approaches for cancers that are difficult to treat [55]. Oncolytic viruses not only selectively kill tumor cells but also trigger protective antitumor immune responses that prevent recurrence [56,57]. This is especially relevant in the case of pancreatic cancers, which are characterized as “cold” tumors due to their highly immunosuppressive microenvironment [58]. Oncolytic viruses have the ability to render the tumor microenvironment “pro-immune” and thus may be able to sensitize pancreatic tumors to immunotherapeutic treatments [57]. In this study, we were able to successfully achieve the oncolytic virus infection of EUS-FNA patient-derived xenografts in all attempted cases, suggesting that these models can be directly applied to the evaluation of the unique susceptibility of patients to oncolytic virus infection. The pancreatic xenograft biobank that we have established is currently being used for ancillary studies to further characterize pancreatic cancer subtypes and treatments.

Our study had some limitations. Firstly, due to sequencing volume and sample availability constrains, we were only able to sequence a select set of normal tissue samples for genetic comparisons. Future studies from our group will aim to confirm our findings with a larger number of control genetic samples. Secondly, while here we demonstrate proof of principle for the use of oncolytic viruses in the context of PDXs, as PDXs are grown in immunodeficient animals, these models are not representative of the native immune conditions in the corresponding patient. The work of our group centers on continuously improving oncolytic virus delivery and longevity in the context of an intact immune response. Therefore, we expect that findings from PDX oncolytic virus infections could be, in the future, combined with novel immune-escape methodologies for the application of personalized OV therapeutics.

## 5. Conclusions

EUS-FNA is a suitable, minimally invasive technique for acquiring adequate tissue for genetic studies, which will likely become the standard of care for personalized medicine in the treatment of pancreatic cancer in the near future. Importantly, it can be applied to xenograft generation for non-resectable pancreatic tumors, which are found in more than 80% of pancreatic cancer cases. The techniques reviewed here could be used for other cancers requiring peri-treatment molecular characterization and xenotransplant animal models and could be applied to a variety of personalized treatment therapies, including oncolytic virus-based sensitization to immunotherapy.

## Figures and Tables

**Table 1:** Patient characteristics.

Variables	Value	%
**Demographics**		
Gender	M—19	63.3
	F—11	36.7
Average Age	M: 65.1	NA
	F: 75.5	NA
**Symptoms**		
Weight Loss	Y—25	83
	N—5	17
Jaundice	Y—9	30
	N—21	70
Abdominal Pain	Y—23	77
	N—7	23
**Health History**		
Previous Cancer Diagnosis	Y—4	13
	N—26	87
Familial Pancreatic Cancer History	Y—3	10
	N—27	90
If Yes: Associated degree	First Degree—1	33.3
	Second Degree—2	66.6
	Third Degree—0	0
Acute Pancreatitis	Y—1	3
	N—29	97
Diabetic	Y—7	23
	N—23	77
**Lifestyle Factors**		
Smoking History	Y—6	20
	N—24	80
Alcohol Consumption	Y—14	47
	N—16	53
**Disease Risk Category**		
Low Risk	11	37
Increased Risk	4	13
High Risk	0	0
No Risk	15	50
**Disease Characteristics**		
Stage	Locally advanced—27	90
	Metastatic—3	10
Tumor Type	Adenocarcinoma—29	96.7
	Acinar cell carcinoma—1	3.3
Tumor Characteristics	Well differentiated—18	60
	Moderately differentiated—10	33.3
	Poorly differentiated—2	6.7
**Outcomes**		
Adverse events	None—30	100
	Concerns—0	0
Clinical Outcome	Surgery—2	6.6
	Chemotherapy—19	63.3
	Radiation—0	0
	No therapy—10	33.3

**Table 2:** Identified genetic variations likely to be somatic.

Chromosome	Position	Ref	Alt	Prediction	Gene	Consequence	Times Called	PolyPhen-2		SIFT	
chr3	10188328	C	T	Likely Somatic	*VHL*	intronic	1	Not found	N/A	Not found	N/A
chr3	178952085	A	G	Likely Somatic	*PIK3CA*	nonsynonymous SNV *	1	Not found	N/A	0.136	Borderline
chr3	37083758	G	C	Potentially Somatic	*MLH1*	splicing	1	Not found	N/A	Not found	N/A
chr3	37090070	G	T	Likely Somatic	*MLH1*	synonymous SNV	3	Not found	N/A	1	Tolerant
chr3	37092176	C	T	Potentially Somatic	*MLH1*	UTR3	1	Not found	N/A	Not found	N/A
chr4	1805639	A	G	Potentially Somatic	*FGFR3*	intronic	1	Not found	N/A	Not found	N/A
chr4	55161348	T	A	Potentially Somatic	*PDGFRA*	nonsynonymous SNV	1	Not found	N/A	Not found	N/A
chr4	55593481	A	G	Likely Somatic	*KIT*	synonymous SNV	1	Not found	N/A	1	Tolerant
chr5	112174456	A	T	Potentially Somatic	*APC*	synonymous SNV	2	Not found	N/A	1	Tolerant
chr5	112175240	G	C	Likely Somatic	*APC*	nonsynonymous SNV	1	0.001	Benign	0.11	Borderline
chr7	116339642	G	T	Likely Somatic	*MET*	nonsynonymous SNV	1	Not found	N/A	0.16	Borderline
chr7	116411990	C	T	Likely Somatic	*MET*	nonsynonymous SNV	1	0.999	Probably damaging	0.03	Intolerant
chr7	140453136	A	C	Likely Somatic	*BRAF*	nonsynonymous SNV	1	Not found	N/A	Not found	N/A
chr7	140453136	A	T	Likely Somatic	*BRAF*	nonsynonymous SNV	1	Not found	N/A	Not found	N/A
chr7	140453137	C	T	Likely Somatic	*BRAF*	nonsynonymous SNV	1	Not found	N/A	0.02	Intolerant
chr7	140481393	T	C	Likely Somatic	*BRAF*	nonsynonymous SNV	1	Not found	N/A	Not found	N/A
chr7	55268949	A	G	Potentially Somatic	*EGFR*	synonymous SNV	1	Not found	N/A	1	Tolerant
chr8	42174380	G	A	Potentially Somatic	*IKBKB*	synonymous SNV	1	Not found	N/A	1	Tolerant
chr9	139397728	C	T	Potentially Somatic	*NOTCH1*	synonymous SNV	1	Not found	N/A	0.563	Tolerant
chr10	43597827	C	A	Potentially Somatic	*RET*	synonymous SNV	1	Not found	N/A	1	Tolerant
chr10	89690764	A	G	Likely Somatic	*PTEN*	intronic	1	Not found	N/A	Not found	N/A
chr12	25380275	T	G	Likely Somatic	*KRAS*	nonsynonymous SNV	1	0.142	Benign	0.002	Intolerant
chr12	25398284	C	A	Likely Somatic	*KRAS*	nonsynonymous SNV	8	Not found	N/A	Not found	N/A
chr12	25398284	C	T	Likely Somatic	*KRAS*	nonsynonymous SNV	4	Not found	N/A	Not found	N/A
chr12	25398285	C	A	Likely Somatic	*KRAS*	nonsynonymous SNV	1	Not found	N/A	Not found	N/A
chr12	25398285	C	G	Likely Somatic	*KRAS*	nonsynonymous SNV	5	Not found	N/A	Not found	N/A
chr13	32912008	G	A	Potentially Somatic	*BRCA2*	synonymous SNV	1	Not found	N/A	1	Tolerant
chr17	12028657	G	A	Likely Somatic	*MAP2K4*	nonsynonymous SNV	1	Not found	N/A	Not found	N/A
chr17	7577093	C	G	Likely Somatic	*TP53*	nonsynonymous SNV	1	Not found	N/A	Not found	N/A
chr17	7577120	C	T	Likely Somatic	*TP53*	nonsynonymous SNV	1	0.985	Probably damaging	Not found	N/A
chr17	7577124	C	T	Likely Somatic	*TP53*	nonsynonymous SNV	1	Not found	N/A	Not found	N/A
chr17	7577127	C	A	Likely Somatic	*TP53*	stopgain SNV	1	Not found	N/A	Not found	N/A
chr17	7577130	A	G	Likely Somatic	*TP53*	nonsynonymous SNV	1	Not found	N/A	Not found	N/A
chr17	7577548	C	T	Likely Somatic	*TP53*	nonsynonymous SNV	2	1	Probably damaging	Not found	N/A
chr17	7578190	T	C	Likely Somatic	*TP53*	nonsynonymous SNV	1	Not found	N/A	Not found	N/A
chr17	7578217	G	A	Likely Somatic	*TP53*	nonsynonymous SNV	1	Not found	N/A	Not found	N/A
chr17	7578263	G	A	Likely Somatic	*TP53*	stopgain SNV	1	Not found	N/A	Not found	N/A
chr17	7578406	C	T	Likely Somatic	*TP53*	nonsynonymous SNV	1	0.881	Possibly damaging	Not found	N/A
chr18	48575202	C	G	Potentially Somatic	*SMAD4*	nonsynonymous SNV	1	Not found	N/A	Not found	N/A
chr21	36259324	A	G	Likely Somatic	*RUNX1*	nonsynonymous SNV	2	Not found	N/A	>0.4	Tolerant
chr22	30067871	G	A	Potentially Somatic	*NF2*	synonymous SNV	1	Not found	N/A	>0.3	Tolerant
chr22	30077428	A	G	Potentially Somatic	*NF2*	synonymous SNV	1	Not found	N/A	Not found	N/A

* Non-synonymous and stopgain single-nucleotide variants (SNVs) are shaded.

**Table 3:** Somatic variations identified in primary tumors and P1 xenografts, as confirmed by genotyping of normal control.

Type of Variant	Gene	Chromosome	Position	Reference	Variant	Consequence	PolyPhen-2		SIFT	
Somatic	*KRAS*	chr12	25398284	C	A	Nonsynonymous single-nucleotide variation	Not found	N/A	Not found	N/A
Somatic	*KRAS*	chr12	25398284	C	T	Nonsynonymous single-nucleotide variation	Not found	N/A	Not found	N/A
Somatic	*KRAS*	chr12	25398285	C	G	Nonsynonymous single-nucleotide variation	Not found	N/A	Not found	N/A
Somatic	*TP53*	chr17	7578406	C	T	Nonsynonymous single-nucleotide variation	0.881	Possibly damaging	Not found	N/A

## Data Availability

Data are contained within the article.

## Supplementary Materials

The following supporting information can be downloaded at https://www.mdpi.com/article/10.3390/cancers16152721/s1, Table S1: Percent tumor area for select P1 and P2 xenografts.
