# Supplementary material for: Molecular Characterization and Xenotransplantation of Pancreatic Cancer Using Endoscopic Ultrasound-Guided Fine Needle Aspiration (EUS-FNA)
Source: Cancers (Basel). 2024 Jul 31;16(15):2721. doi: 10.3390/cancers16152721 (PMC11311391; doi:10.3390/cancers16152721)
Supplement: Supplementary file 1 [file cancers-16-02721-s001.zip › cancers-3050894-supplementary.pdf]

Supplementary Table S1: Percent tumor area for select P1 and P2 xenografts.

Xenograft (patient # -passage #)	Tumour Type	Amount of Tumour Occupying the Section (%)	Xenograft (patient # -passage #)	Tumour Type	Amount of Tumour Occupying the Section (%)
P003-P1	Adenocarcinoma	80	P006-P2	Adenocarcinoma	95
P006-P1	Adenocarcinoma	90	P008-P2	Adenocarcinoma	90
P008-P1	Adenocarcinoma	90	P009-P2	Adenocarcinoma	40
P009-P1	Adenocarcinoma	50	P014-P2	Adenocarcinoma	60
P010-P1	Adenocarcinoma	40	P015-P2	Adenocarcinoma	60
P013-P1	Adenocarcinoma	35	P021-P2	Adenocarcinoma	40
P015-P1	Adenocarcinoma	95	P023-P2	Adenocarcinoma	70
P017-P1	Adenocarcinoma	80			
P019-P1	Adenocarcinoma	70			
P020-P1	Neuroendocrine vs acinic cell	95			
P021-P1	Adenocarcinoma	50			
P025-P1	Adenocarcinoma	75			
P027-P1	Adenocarcinoma	85			
P029-P1	Adenocarcinoma	70			
P033-P1	Adenocarcinoma	90			
P034-P1	Adenocarcinoma	80			
P036-P1	Adenocarcinoma	60			
P037-P1	Adenocarcinoma	80			
